# 4-Amino-2-hy­droxy­benzohydrazide

**DOI:** 10.1107/S1600536812026190

**Published:** 2012-06-16

**Authors:** Hadi Kargar, Reza Kia, Muhammad Nawaz Tahir

**Affiliations:** a Department of Chemistry, Payame Noor University, PO Box 19395-3697 Tehran, I.R. of IRAN; b Department of Chemistry, Science and Research Branch, Islamic Azad University, Tehran, Iran; c Department of Physics, University of Sargodha, Punjab, Pakistan

## Abstract

The asymmetric unit of the title compound, C_7_H_9_N_3_O_2_, comprises two crystallographically independent mol­ecules (*A* and *B*). In each mol­ecule there is an intra­molecular O—H⋯O hydrogen bond making an *S*(6) ring motif. In the crystal, a pair of N—H⋯N hydrogen bonds link the two mol­ecules (*A* and *B*) into a dimer with an *R* ^2^_2_(6) ring motif. The *B* mol­ecules are linked *via* pairs of N—H⋯O hydrogen bonds, forming inversion dimers with an *R* ^2^_2_(10) ring motif. The mol­ecules are further linked *via* other N—H⋯O hydrogen bonds, forming undulating two-dimensional networks lying parallel to the *bc* plane. These networks are finally linked *via* N—H⋯O hydrogen bonds, forming a three-dimensional structure.

## Related literature

For background to Schiff bases derived from benzohydrazide, see: Xu (2012 ▶); Bakir & Green (2002 ▶). For hydrogen-bond motifs, see: Bernstein *et al.* (1995 ▶). For standard bond lengths, see: Allen *et al.* (1987 ▶).
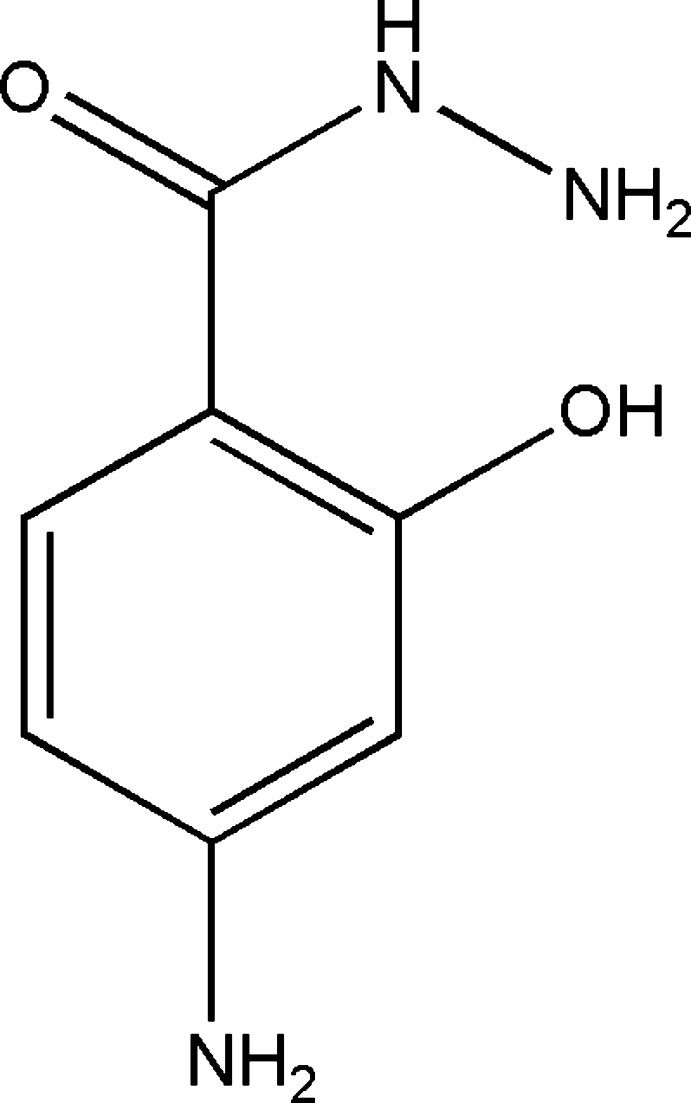


## Experimental

### 

#### Crystal data

C_7_H_9_N_3_O_2_
*M* *_r_* = 167.17
Monoclinic, 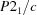

*a* = 5.6424 (3) Å
*b* = 18.3221 (12) Å
*c* = 14.7164 (9) Å
β = 94.087 (3)°
*V* = 1517.52 (16) Å^3^
*Z* = 8
Mo *K*α radiation
μ = 0.11 mm^−1^
*T* = 291 K
0.32 × 0.16 × 0.14 mm

#### Data collection

Bruker SMART APEXII CCD area-detector diffractometer
Absorption correction: multi-scan (*SADABS*; Bruker, 2005 ▶) *T*_min_ = 0.965, *T*_max_ = 0.985
12530 measured reflections
3366 independent reflections
2092 reflections with *I* > 2σ(*I*)
*R*_int_ = 0.027

#### Refinement

*R*[*F* ^2^ > 2σ(*F* ^2^)] = 0.045
*wR*(*F* ^2^) = 0.133
*S* = 1.01
3366 reflections
219 parameters
H-atom parameters constrained
Δρ_max_ = 0.21 e Å^−3^
Δρ_min_ = −0.21 e Å^−3^

### 

Data collection: *APEX2* (Bruker, 2005 ▶); cell refinement: *SAINT* (Bruker, 2005 ▶); data reduction: *SAINT*; program(s) used to solve structure: *SHELXS97* (Sheldrick, 2008 ▶); program(s) used to refine structure: *SHELXL97* (Sheldrick, 2008 ▶); molecular graphics: *SHELXTL* (Sheldrick, 2008 ▶)’; software used to prepare material for publication: *SHELXTL* and *PLATON* (Spek, 2009 ▶).

## Supplementary Material

Crystal structure: contains datablock(s) global, I. DOI: 10.1107/S1600536812026190/su2448sup1.cif

Structure factors: contains datablock(s) I. DOI: 10.1107/S1600536812026190/su2448Isup2.hkl

Supplementary material file. DOI: 10.1107/S1600536812026190/su2448Isup3.cml

Additional supplementary materials:  crystallographic information; 3D view; checkCIF report

## Figures and Tables

**Table 1:** Hydrogen-bond geometry (Å, °)

*D*—H⋯*A*	*D*—H	H⋯*A*	*D*⋯*A*	*D*—H⋯*A*
O1—H1⋯O2	0.82	1.80	2.5297 (18)	147
O3—H3⋯O4	0.82	1.80	2.528 (2)	147
N2—H2⋯N6^i^	0.98	2.07	2.958 (2)	149
N5—H5⋯N3^ii^	0.99	2.04	2.934 (2)	150
N6—H1*N*6⋯O4^iii^	0.91	2.25	2.9424 (18)	133
N1—H1*B*⋯O1^iv^	0.86	2.24	3.0358 (19)	154
N4—H4*B*⋯O2^v^	0.86	2.30	2.974 (2)	136
N6—H2*N*6⋯O3^ii^	0.96	2.54	3.207 (2)	127

Symmetry codes: (i) 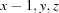; (ii) 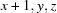; (iii) 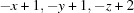; (iv) 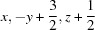; (v) 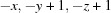.

**Fig. 1.**
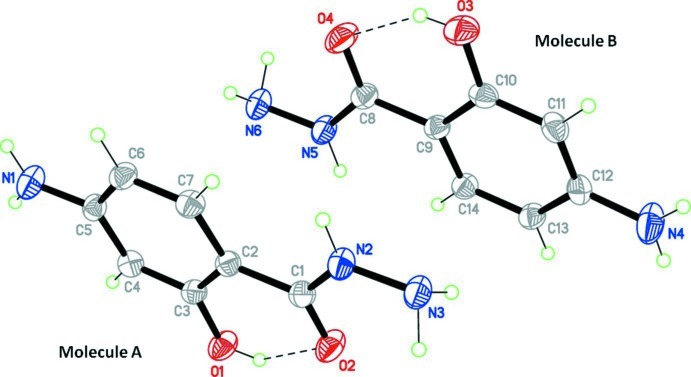
A view of the molecular structure of the two independent molecules of the title compound, showing 40% probability displacement ellipsoids and the atomic numbering. The dashed lines shows the intramolecular O-H···O hydrogen bonds (see Table 1 for details).

**Fig. 2.**
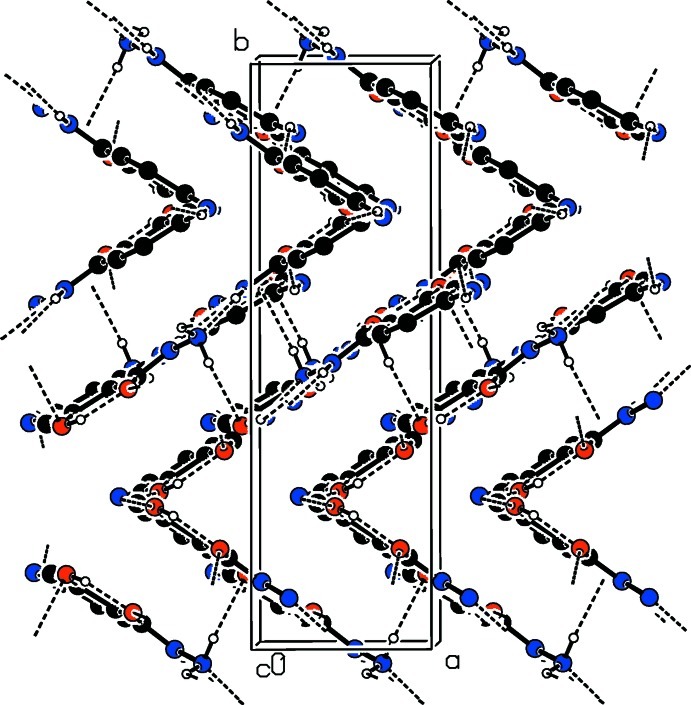
A view along the c axis of crystal packing of the title compound, showing the two-dimensional networks formed through N—H···O and N—H···N hydrogen bonds (dashed lines; see Table 1 for details). Only the H atoms involved in the interactions are shown.

## supplementary crystallographic information

### Comment

Structures of Schiff bases derived from substituted 4-aminobenzohydrazide closely related to the title compound have been reported earlier (Xu, 2012; Bakir & Green, 2002). In order to explore new Schiff base compounds of hydroxy derivatives of 4-aminobenzohydrazide, the title compound was prepared and we report herein on its crystal structure.

The asymmetric unit of the title compound, Fig. 1, comprises two crystallographically independent molecules (A and B). The bond lengths (Allen *et al.*, 1987) and angles are within normal ranges. In each molecule an intramolecular O—H···O hydrogen bond (Table 1) make an *S(6)* ring motif (Bernstein *et al.*, 1995).

In the crystal, there are number of N-H···N and N-H..O hydrogen bonds linking the molecules (Table 1). A pair of N—H···N hydrogen bonds link the two molecules (A and B) to form dimers with an *R^2^_2_(6)* ring motif. The B molecules are linked via pairs of N—H···O hydrogen bonds to form inversion dimers with an *R^2^_2_(10)* ring motif. The molecules are further linked through other N—H···O hydrogen bonds forming undulating two-dimensional networks lying parallel to the *bc* plane. These networks are finally linked via an N-H···O hydrogen bond to form a three-dimensional structure (Fig. 2).

### Experimental

The title compound was synthesized by adding 1 mmol of methyl 4-amino-2-hydroxybenzoate to a solution of hydrazine hydrate (80%) (1 mmol) in methanol (30 ml). The mixture was refluxed with stirring for 30 min and after cooling to room temperature a white precipitate was filtered off and washed with diethylether and dried in air. Colourless needle-like crystals of the title compound, suitable for *X*-ray structure analysis, were recrystallized from ethanol by slow evaporation of the solvents at room temperature over several days.

### Refinement

The N-bound H-atoms were located in a difference Fourier map and were constrained to ride on their parent N atoms with U_iso_(H) = 1.2U_eq_(N). The OH and C-bound H atoms were included in calculated positions and treated as riding atoms: O-H = 0.82 Å, C-H = 0.93 Å, with U_iso_(H) = k × U_eq_(O,C) where k = 1.5 for OH H atoms and = 1.2 for other H atoms.

### Crystal data

**Table:**

C_7_H_9_N_3_O_2_	*F*(000) = 704
*M**_r_* = 167.17	*D*_x_ = 1.463 Mg m^−^^3^
Monoclinic, *P*2_1_/*c*	Mo *K*α radiation, λ = 0.71073 Å
Hall symbol: -P 2ybc	Cell parameters from 2540 reflections
*a* = 5.6424 (3) Å	θ = 2.5–27.4°
*b* = 18.3221 (12) Å	µ = 0.11 mm^−^^1^
*c* = 14.7164 (9) Å	*T* = 291 K
β = 94.087 (3)°	Needle, colourless
*V* = 1517.52 (16) Å^3^	0.32 × 0.16 × 0.14 mm
*Z* = 8	

### Data collection

**Table:**

Bruker SMART APEXII CCD area-detector diffractometer	3366 independent reflections
Radiation source: fine-focus sealed tube	2092 reflections with *I* > 2σ(*I*)
Graphite monochromator	*R*_int_ = 0.027
φ and ω scans	θ_max_ = 27.2°, θ_min_ = 1.8°
Absorption correction: multi-scan (*SADABS*; Bruker, 2005)	*h* = −7→7
*T*_min_ = 0.965, *T*_max_ = 0.985	*k* = −23→23
12530 measured reflections	*l* = −18→18

### Refinement

**Table:**

Refinement on *F*^2^	Primary atom site location: structure-invariant direct methods
Least-squares matrix: full	Secondary atom site location: difference Fourier map
*R*[*F*^2^ > 2σ(*F*^2^)] = 0.045	Hydrogen site location: inferred from neighbouring sites
*wR*(*F*^2^) = 0.133	H-atom parameters constrained
*S* = 1.01	*w* = 1/[σ^2^(*F*_o_^2^) + (0.0609*P*)^2^ + 0.2376*P*] where *P* = (*F*_o_^2^ + 2*F*_c_^2^)/3
3366 reflections	(Δ/σ)_max_ < 0.001
219 parameters	Δρ_max_ = 0.21 e Å^−^^3^
0 restraints	Δρ_min_ = −0.21 e Å^−^^3^

### Special details

**Table:**

Geometry. All esds (except the esd in the dihedral angle between two l.s. planes) are estimated using the full covariance matrix. The cell esds are taken into account individually in the estimation of esds in distances, angles and torsion angles; correlations between esds in cell parameters are only used when they are defined by crystal symmetry. An approximate (isotropic) treatment of cell esds is used for estimating esds involving l.s. planes.
Refinement. Refinement of F^2^ against ALL reflections. The weighted R-factor wR and goodness of fit S are based on F^2^, conventional R-factors R are based on F, with F set to zero for negative F^2^. The threshold expression of F^2^ > 2sigma(F^2^) is used only for calculating R-factors(gt) etc. and is not relevant to the choice of reflections for refinement. R-factors based on F^2^ are statistically about twice as large as those based on F, and R- factors based on ALL data will be even larger.

### Fractional atomic coordinates and isotropic or equivalent isotropic displacement parameters (Å^2^)

**Table:**

	*x*	*y*	*z*	*U*_iso_*/*U*_eq_	
C1	0.1395 (3)	0.65395 (10)	0.73974 (11)	0.0391 (4)	
C2	0.2934 (3)	0.68029 (9)	0.81762 (11)	0.0364 (4)	
C3	0.4957 (3)	0.72159 (9)	0.80167 (11)	0.0369 (4)	
C4	0.6503 (3)	0.74457 (10)	0.87301 (11)	0.0398 (4)	
H4	0.7847	0.7712	0.8608	0.048*	
C5	0.6085 (3)	0.72868 (9)	0.96218 (11)	0.0386 (4)	
C6	0.4041 (3)	0.68873 (10)	0.97971 (11)	0.0430 (4)	
H6	0.3718	0.6781	1.0394	0.052*	
C7	0.2524 (3)	0.66546 (10)	0.90872 (11)	0.0414 (4)	
H7	0.1179	0.6390	0.9213	0.050*	
C8	0.3586 (3)	0.46432 (10)	0.83167 (11)	0.0411 (4)	
C9	0.2091 (3)	0.44085 (9)	0.75099 (11)	0.0363 (4)	
C10	0.0013 (3)	0.40061 (10)	0.76272 (11)	0.0402 (4)	
C11	−0.1501 (3)	0.38160 (10)	0.68907 (12)	0.0442 (5)	
H11	−0.2893	0.3564	0.6985	0.053*	
C12	−0.0989 (3)	0.39927 (10)	0.60123 (12)	0.0428 (4)	
C13	0.1119 (3)	0.43658 (10)	0.58807 (11)	0.0415 (4)	
H13	0.1520	0.4475	0.5294	0.050*	
C14	0.2596 (3)	0.45715 (10)	0.66168 (11)	0.0405 (4)	
H14	0.3978	0.4827	0.6518	0.049*	
N1	0.7591 (3)	0.75402 (10)	1.03293 (10)	0.0567 (5)	
H1A	0.8806	0.7800	1.0218	0.068*	
H1B	0.7317	0.7438	1.0883	0.068*	
N2	−0.0459 (3)	0.61215 (8)	0.75474 (10)	0.0445 (4)	
H2	−0.0935	0.5975	0.8151	0.053*	
N3	−0.2002 (3)	0.58528 (9)	0.68260 (10)	0.0486 (4)	
H1N3	−0.1141	0.5589	0.6368	0.058*	
H2N3	−0.2503	0.6231	0.6408	0.058*	
N4	−0.2532 (3)	0.38142 (11)	0.52828 (11)	0.0686 (6)	
H4A	−0.3835	0.3590	0.5369	0.082*	
H4B	−0.2195	0.3927	0.4740	0.082*	
N5	0.5410 (3)	0.50862 (9)	0.81992 (9)	0.0468 (4)	
H5	0.5880	0.5239	0.7595	0.056*	
N6	0.6986 (3)	0.53248 (10)	0.89273 (9)	0.0519 (4)	
H1N6	0.6126	0.5492	0.9381	0.062*	
H2N6	0.7604	0.4892	0.9220	0.062*	
O1	0.5463 (2)	0.74076 (8)	0.71656 (8)	0.0523 (4)	
H1	0.4410	0.7260	0.6799	0.078*	
O2	0.1813 (2)	0.66956 (8)	0.65963 (8)	0.0560 (4)	
O3	−0.0582 (2)	0.37996 (8)	0.84652 (8)	0.0566 (4)	
H3	0.0483	0.3916	0.8846	0.085*	
O4	0.3139 (3)	0.44440 (8)	0.90981 (8)	0.0619 (4)	

### Atomic displacement parameters (Å^2^)

**Table:**

	*U*^11^	*U*^22^	*U*^33^	*U*^12^	*U*^13^	*U*^23^
C1	0.0420 (10)	0.0386 (10)	0.0370 (10)	0.0033 (8)	0.0057 (8)	−0.0009 (8)
C2	0.0371 (9)	0.0374 (10)	0.0347 (9)	0.0031 (8)	0.0033 (7)	−0.0009 (7)
C3	0.0398 (10)	0.0382 (10)	0.0335 (9)	0.0054 (8)	0.0086 (7)	0.0005 (7)
C4	0.0356 (9)	0.0423 (10)	0.0420 (10)	−0.0012 (8)	0.0076 (8)	−0.0012 (8)
C5	0.0393 (10)	0.0406 (10)	0.0358 (9)	0.0065 (8)	0.0012 (7)	−0.0061 (8)
C6	0.0486 (11)	0.0490 (11)	0.0319 (9)	0.0020 (9)	0.0069 (8)	0.0009 (8)
C7	0.0402 (10)	0.0440 (10)	0.0409 (10)	−0.0023 (8)	0.0085 (8)	0.0026 (8)
C8	0.0438 (10)	0.0449 (11)	0.0349 (9)	0.0046 (9)	0.0038 (8)	0.0002 (8)
C9	0.0392 (9)	0.0370 (10)	0.0328 (9)	0.0022 (8)	0.0043 (7)	0.0016 (7)
C10	0.0474 (10)	0.0355 (10)	0.0389 (10)	0.0015 (8)	0.0111 (8)	0.0040 (8)
C11	0.0444 (11)	0.0407 (11)	0.0485 (11)	−0.0104 (8)	0.0091 (9)	−0.0032 (8)
C12	0.0458 (11)	0.0386 (10)	0.0437 (10)	−0.0017 (9)	0.0018 (8)	−0.0082 (8)
C13	0.0454 (10)	0.0464 (11)	0.0333 (9)	−0.0019 (9)	0.0068 (8)	0.0013 (8)
C14	0.0385 (10)	0.0448 (10)	0.0383 (10)	−0.0038 (8)	0.0040 (8)	0.0028 (8)
N1	0.0553 (10)	0.0755 (12)	0.0390 (9)	−0.0105 (9)	0.0000 (7)	−0.0084 (8)
N2	0.0430 (9)	0.0514 (10)	0.0387 (8)	−0.0073 (7)	0.0012 (7)	−0.0050 (7)
N3	0.0503 (9)	0.0544 (10)	0.0401 (8)	−0.0057 (8)	−0.0036 (7)	−0.0062 (7)
N4	0.0645 (11)	0.0898 (14)	0.0508 (10)	−0.0334 (10)	0.0001 (9)	−0.0121 (9)
N5	0.0470 (9)	0.0578 (10)	0.0352 (8)	−0.0085 (8)	−0.0004 (7)	−0.0034 (7)
N6	0.0514 (9)	0.0648 (11)	0.0386 (8)	−0.0061 (8)	−0.0030 (7)	−0.0081 (7)
O1	0.0553 (9)	0.0687 (9)	0.0332 (7)	−0.0120 (7)	0.0064 (6)	0.0044 (6)
O2	0.0613 (9)	0.0737 (10)	0.0329 (7)	−0.0143 (7)	0.0018 (6)	0.0007 (6)
O3	0.0632 (9)	0.0657 (9)	0.0424 (7)	−0.0113 (8)	0.0128 (6)	0.0108 (7)
O4	0.0692 (10)	0.0838 (11)	0.0324 (7)	−0.0133 (8)	0.0014 (6)	0.0080 (7)

### Geometric parameters (Å, º)

**Table:**

C1—O2	1.252 (2)	C11—C12	1.383 (2)
C1—N2	1.328 (2)	C11—H11	0.9300
C1—C2	1.469 (2)	C12—N4	1.373 (2)
C2—C7	1.403 (2)	C12—C13	1.397 (2)
C2—C3	1.403 (2)	C13—C14	1.372 (2)
C3—O1	1.3506 (19)	C13—H13	0.9300
C3—C4	1.382 (2)	C14—H14	0.9300
C4—C5	1.381 (2)	N1—H1A	0.8600
C4—H4	0.9300	N1—H1B	0.8600
C5—N1	1.377 (2)	N2—N3	1.4125 (19)
C5—C6	1.406 (2)	N2—H2	0.9837
C6—C7	1.371 (2)	N3—H1N3	0.9851
C6—H6	0.9300	N3—H2N3	0.9568
C7—H7	0.9300	N4—H4A	0.8600
C8—O4	1.249 (2)	N4—H4B	0.8600
C8—N5	1.331 (2)	N5—N6	1.4123 (19)
C8—C9	1.471 (2)	N5—H5	0.9858
C9—C14	1.397 (2)	N6—H1N6	0.9064
C9—C10	1.406 (2)	N6—H2N6	0.9561
C10—O3	1.355 (2)	O1—H1	0.8200
C10—C11	1.376 (2)	O3—H3	0.8200
			
O2—C1—N2	119.45 (16)	C10—C11—H11	119.4
O2—C1—C2	121.28 (16)	C12—C11—H11	119.4
N2—C1—C2	119.26 (15)	N4—C12—C11	120.81 (17)
C7—C2—C3	117.12 (15)	N4—C12—C13	120.45 (17)
C7—C2—C1	123.58 (16)	C11—C12—C13	118.74 (16)
C3—C2—C1	119.29 (15)	C14—C13—C12	120.03 (16)
O1—C3—C4	117.51 (16)	C14—C13—H13	120.0
O1—C3—C2	121.56 (15)	C12—C13—H13	120.0
C4—C3—C2	120.93 (15)	C13—C14—C9	122.08 (17)
C5—C4—C3	121.11 (17)	C13—C14—H14	119.0
C5—C4—H4	119.4	C9—C14—H14	119.0
C3—C4—H4	119.4	C5—N1—H1A	120.0
N1—C5—C4	120.65 (17)	C5—N1—H1B	120.0
N1—C5—C6	120.48 (16)	H1A—N1—H1B	120.0
C4—C5—C6	118.83 (15)	C1—N2—N3	121.79 (14)
C7—C6—C5	119.88 (16)	C1—N2—H2	125.2
C7—C6—H6	120.1	N3—N2—H2	113.0
C5—C6—H6	120.1	N2—N3—H1N3	112.2
C6—C7—C2	122.10 (17)	N2—N3—H2N3	111.6
C6—C7—H7	118.9	H1N3—N3—H2N3	93.0
C2—C7—H7	118.9	C12—N4—H4A	120.0
O4—C8—N5	120.42 (16)	C12—N4—H4B	120.0
O4—C8—C9	121.05 (17)	H4A—N4—H4B	120.0
N5—C8—C9	118.51 (15)	C8—N5—N6	122.74 (15)
C14—C9—C10	117.09 (15)	C8—N5—H5	123.3
C14—C9—C8	123.60 (16)	N6—N5—H5	113.7
C10—C9—C8	119.31 (15)	N5—N6—H1N6	108.8
O3—C10—C11	117.79 (16)	N5—N6—H2N6	105.9
O3—C10—C9	121.39 (16)	H1N6—N6—H2N6	98.4
C11—C10—C9	120.82 (15)	C3—O1—H1	109.5
C10—C11—C12	121.17 (17)	C10—O3—H3	109.5
			
O2—C1—C2—C7	178.86 (16)	O4—C8—C9—C10	5.5 (3)
N2—C1—C2—C7	−1.8 (3)	N5—C8—C9—C10	−173.19 (15)
O2—C1—C2—C3	−2.3 (3)	C14—C9—C10—O3	177.62 (16)
N2—C1—C2—C3	177.02 (16)	C8—C9—C10—O3	−3.2 (3)
C7—C2—C3—O1	−177.79 (15)	C14—C9—C10—C11	−3.1 (3)
C1—C2—C3—O1	3.3 (2)	C8—C9—C10—C11	176.03 (16)
C7—C2—C3—C4	1.8 (2)	O3—C10—C11—C12	−178.52 (16)
C1—C2—C3—C4	−177.12 (16)	C9—C10—C11—C12	2.2 (3)
O1—C3—C4—C5	178.53 (16)	C10—C11—C12—N4	−178.66 (17)
C2—C3—C4—C5	−1.1 (3)	C10—C11—C12—C13	0.5 (3)
C3—C4—C5—N1	−177.69 (16)	N4—C12—C13—C14	177.00 (17)
C3—C4—C5—C6	−0.3 (3)	C11—C12—C13—C14	−2.2 (3)
N1—C5—C6—C7	178.29 (16)	C12—C13—C14—C9	1.2 (3)
C4—C5—C6—C7	0.9 (3)	C10—C9—C14—C13	1.4 (3)
C5—C6—C7—C2	−0.1 (3)	C8—C9—C14—C13	−177.66 (16)
C3—C2—C7—C6	−1.2 (3)	O2—C1—N2—N3	−0.7 (3)
C1—C2—C7—C6	177.67 (16)	C2—C1—N2—N3	179.94 (15)
O4—C8—C9—C14	−175.39 (17)	O4—C8—N5—N6	3.1 (3)
N5—C8—C9—C14	5.9 (3)	C9—C8—N5—N6	−178.16 (16)

### Hydrogen-bond geometry (Å, º)

**Table:**

*D*—H···*A*	*D*—H	H···*A*	*D*···*A*	*D*—H···*A*
O1—H1···O2	0.82	1.80	2.5297 (18)	147
O3—H3···O4	0.82	1.80	2.528 (2)	147
N2—H2···N6^i^	0.98	2.07	2.958 (2)	149
N5—H5···N3^ii^	0.99	2.04	2.934 (2)	150
N6—H1*N*6···O4^iii^	0.91	2.25	2.9424 (18)	133
N1—H1*B*···O1^iv^	0.86	2.24	3.0358 (19)	154
N4—H4*B*···O2^v^	0.86	2.30	2.974 (2)	136
N6—H2*N*6···O3^ii^	0.96	2.54	3.207 (2)	127

Symmetry codes: (i) *x*−1, *y*, *z*; (ii) *x*+1, *y*, *z*; (iii) −*x*+1, −*y*+1, −*z*+2; (iv) *x*, −*y*+3/2, *z*+1/2; (v) −*x*, −*y*+1, −*z*+1.
